# Epidemiological changes in oesophageal cancer at National Hospital, Bloemfontein: 1995, 2000 and 2005

**DOI:** 10.4102/phcfm.v2i1.100

**Published:** 2010-06-11

**Authors:** Tian van der Merwe, Ruan van der Walt, Jován Esterhuizen, Stephani Botha, Louis Goedhals, Gina Joubert

**Affiliations:** 1 Faculty of Health Sciences, University of the Free State, South Africa; 2 Department of Oncotherapy, University of the Free State, South Africa; 3 Department of Biostatistics, University of the Free State, South Africa

**Keywords:** cancer, carcinoma, epidemiology, incidence, oesophagus

## Abstract

**Background:**

Oesophageal cancer is a common malignancy with a high mortality rate. The two main histological types are squamous cell and adenocarcinoma. An increase in oesophageal adenocarcinoma has been noted, especially in developed countries.

**Objectives:**

The aim of this retrospective study was to investigate the profile of oesophageal cancer by reviewing medical records of patients diagnosed with oesophageal cancer in 1995, 2000 and 2005.

**Method:**

The study sample consisted of 474 files of patients diagnosed, for the first time, with oesophageal cancer in 1995, 2000 and 2005, at the National Hospital in Bloemfontein and the outreach clinics in surrounding areas. Information reviewed from patient files included: age, race and gender of the patient, as well as topography, size, histological grade and type of the tumour.

**Results:**

The number of newly diagnosed cases of oesophageal carcinoma decreased over the 10-year period. The mean age of patients was > 57 years. The majority of cases were Black patients: 90.5% in 1995, 93.2% in 2000 and 87.7% in 2005. More male patients were seen (71.5% in 1995, 70.1% in 2000 and 64.2% in 2005), although the number of female patients diagnosed with this malignancy increased by 7.3% from 1995 to 2005. The mid- and lower third of the oesophagus were affected most commonly, most lesions were 6 cm – 10 cm in length and classified as Grade II, moderately differentiated tumours. Squamous cell carcinoma was diagnosed in 76.9% of patients in 1995, 90.5% in 2000 and 94.3% in 2005.

**Conclusion:**

The number of newly diagnosed cases of oesophageal carcinoma decreased over the 10-year period, but demographic and disease characteristics remained constant.

## INTRODUCTION

Two histological types of oesophageal cancer that occur most frequently are squamous cell carcinoma and adenocarcinoma. Squamous cell carcinoma originates from the epithelium lining the oesophagus, while the malignant epithelial cells in adenocarcinoma are arranged in a glandular formation. In the past, squamous cell carcinoma occurred more frequently, but, over the past couple of decades, an increase in adenocarcinoma of the oesophagus has been noted.^1^ However, no reports of previous studies explaining this observation could be found in the literature.

Huang^2^ classified the natural history of oesophageal cancer into four different phases. The initial phase of oesophageal carcinoma stretches over an extended period, probably 20 years or more. The first changes that occur are small to moderate hyperplasia of the basal epithelial cells of the oesophageal mucosa. This is followed by the developmental phase in which cancerous cells develop. The developmental phase is clinically latent and may be present for a long period of time. These cells penetrate the basal layer of the epithelium and develop into infiltrating cancer that occurs as a granular plaque. Thereafter, the disease progresses to the overt phase, which is the main clinical phase that includes stages two and three of oesophagus carcinoma. In some cases, the primary cancer enlarges from the originally small, localised, early lesion. This lesion penetrates the deep layers of the oesophageal wall during the process of development. The primary cancer can also develop from a superficial lesion and increase rapidly in size, with penetration of the deeper layers of the oesophagus. The final phase of oesophageal carcinoma is usually short, with extensive extra-oesophageal invasion to the vital organs, for example, metastasis to the lungs.^2^

Three screening modalities are available for early identification of oesophageal cancer: routine double contrast radiology, fibre-optic endoscopy, and oesophageal cytology. Oesophageal endoscopy employs the brush or balloon method, which has been found to show 95% accuracy in the identification of oesophageal cancer. The combination of balloon cytology and fibre-optic endoscopy provides a solid foundation for the early diagnosis of dysplasia and premalignant lesions.^2^

The diagnosis of oesophageal cancer is often made late, when screening is no longer beneficial. In countries with a high incidence of oesophagus carcinoma, such as China, population screening programmes may be employed. In Western countries, however, where the incidence is usually lower, these screening programmes are not always applied.^2^

Different factors are associated with oesophageal cancer and various studies across the world have been conducted to investigate these associations. A patient's ethnicity plays an important role, which is illustrated by the drastic difference between the incidence of oesophageal cancer in Black and White ethnic groups.^3^ Oesophageal cancer in Black South Africans was very rare until the last couple of decades.^4^ In South African Black men, for example, the incidence of oesophageal cancer is 40.9 per 100 000 males, compared to 4.4 per 100 000 males in Mozambique and 1.5 per 100 000 males in Nigeria.^5^

According to a case-control study performed in Soweto, South Africa, involving 200 oesophageal cancer patients and 391 hospital controls, pipe tobacco and the consumption of traditional beer were identified as risk factors for rural Black populations.^4^ Although other research supported tobacco smoking as a risk factor,^6^ a study conducted in Sweden found that alcohol consumption does not have an effect on the development of oesophageal carcinoma.^7^

Several studies were performed to determine whether food types have an effect on the incidence of oesophageal carcinoma. It was found that people consuming insufficient amounts of fruit or cereals,^7^ vegetables^2^ or excessive amounts of meat,^8^ were at greater risk of oesophageal cancer. Chronic malnutrition was also found to increase the risk of oesophageal carcinoma.^3^

A case-control study conducted in Sweden between 1995 and 1997, found that first degree family members did not have an increased risk of cancer in both adenocarcinoma and squamous cell oesophageal carcinoma cases.^9^ On the contrary, a British study on the incidence of oesophageal carcinoma found that heredity did, in fact, play a role, albeit to a minor extent.^7^

The aim of this retrospective study was to investigate the profile of oesophageal cancer by reviewing medical records of patients diagnosed with oesophageal cancer in 1995, 2000 and 2005.

## METHOD

A retrospective analytical study was performed to review medical records for the years 1995, 2000 and 2005. Four hundred and seventy-four files of patients diagnosed with oesophageal cancer for the first time in these years, at the Department of Oncotherapy at the National Hospital in Bloemfontein and outreach clinics in Bethlehem, Kimberley, Kroonstad and Welkom, were included in the study. Files were made available by the Biostatistics Division of the National Hospital. Information regarding the demography of patients, as well as the topography and morphology of the cancer, were obtained from the patients’ files and recorded in a data sheet.

Analysis of data was performed by the Department of Biostatistics, University of the Free State, using SAS software.^10^ Results of categorical variables were summarised as frequencies and percentages, and numerical variables as means, standard deviations or percentiles. The three different years (1995, 2000 and 2005) were compared by means of analysis of variance, Fischer's exact or chi-square tests, as appropriate.

The research protocol was approved by the Ethics Committee of the Faculty of Health Sciences, University of the Free State (ETOVS Stud. Nr. 26/07). Permission was also obtained from the Head of Clinical Services of the Universitas Academic Complex, which includes the National Hospital. Data obtained remained confidential, patients’ anonimity being preserved through indicating only their oncotherapy reference number on the data form.

A pilot study was performed on 20 patient files from 1998 in order to assess certain aspects of the study, such as the availability of resources (funds and personnel), the amount of time required to complete the data sheet, and the executability of each step of the study. The pilot study also provided an indication of possible results that were to be anticipated.

## RESULTS

The number of patients diagnosed with oesophageal carcinoma for the first time decreased across the study period. In 1995, 221 cases were diagnosed, in 2000, 147 cases and in 2005, 106 cases. The mean age of patients was 57.8 years and 57.5 years in 1995 and 2000, respectively, while a slight increase in the mean age (59.6 years) was observed in 2005 (*p* = 0.2758).

Male patients were in the majority over the entire period of time, with 71.5%, 70.1% and 64.2% of patients being male in 1995, 2000 and 2005, respectively. A slight increase in female patients was noted over the 10-year period, with 28.5% of patients being female in 1995, 29.9% in 2000 and 35.8% in 2005.

**FIGURE 1:**
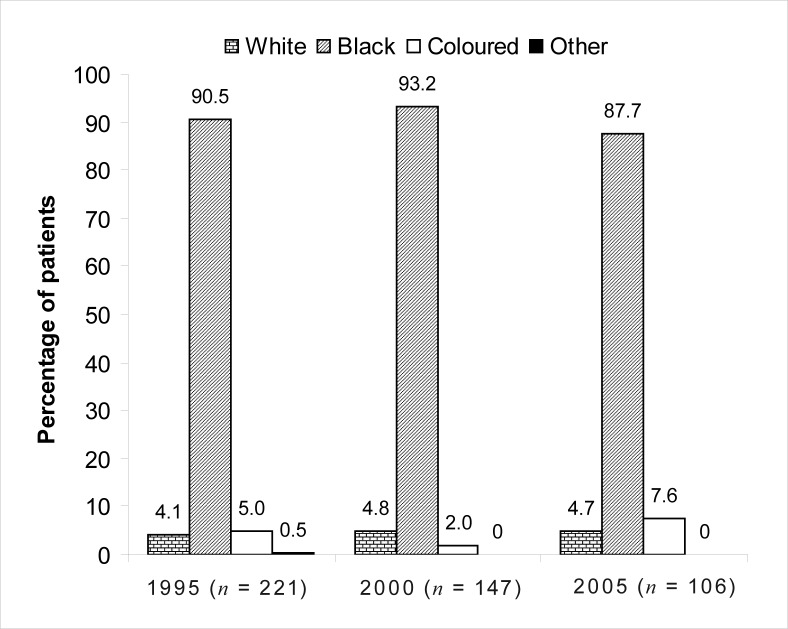
Racial distribution of patients diagnosed with oesophageal cancer in 1995, 2000 and 2005

The vast majority of patients diagnosed with oesophageal cancer were Black (90.5% in 1995, 93.2% in 2000 and 87.7% in 2005). Results of patient distribution according to race are shown in Figure 1.

**FIGURE 2:**
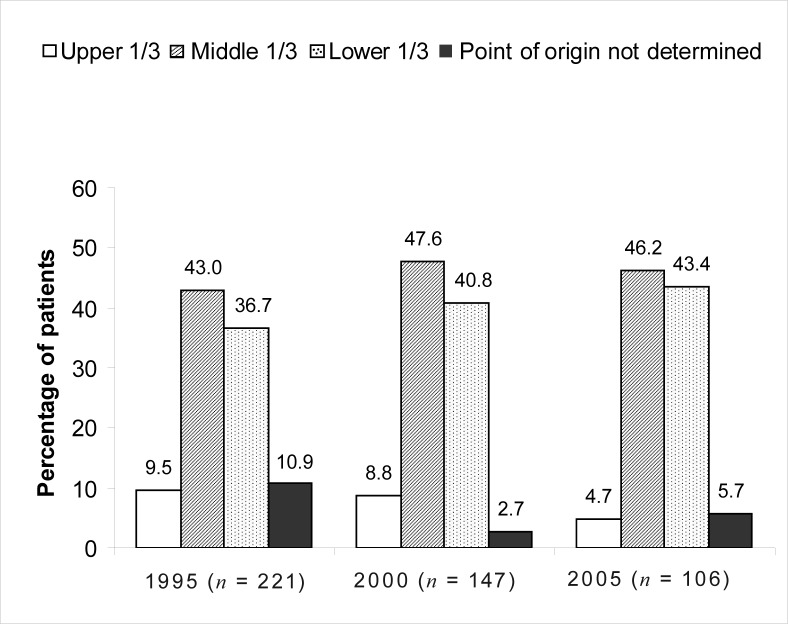
Topographic distribution of oesophageal cancer in 1995, 2000 and 2005

The results showed that the parts of the oesophagus most commonly affected by cancer were the middle and lower third, while involvement of the upper third of the oesophagus remained below 10% for the three years investigated. The topographic distribution of oesophageal cancer in 1995, 2000 and 2005 is shown in Figure 2.

**FIGURE 3:**
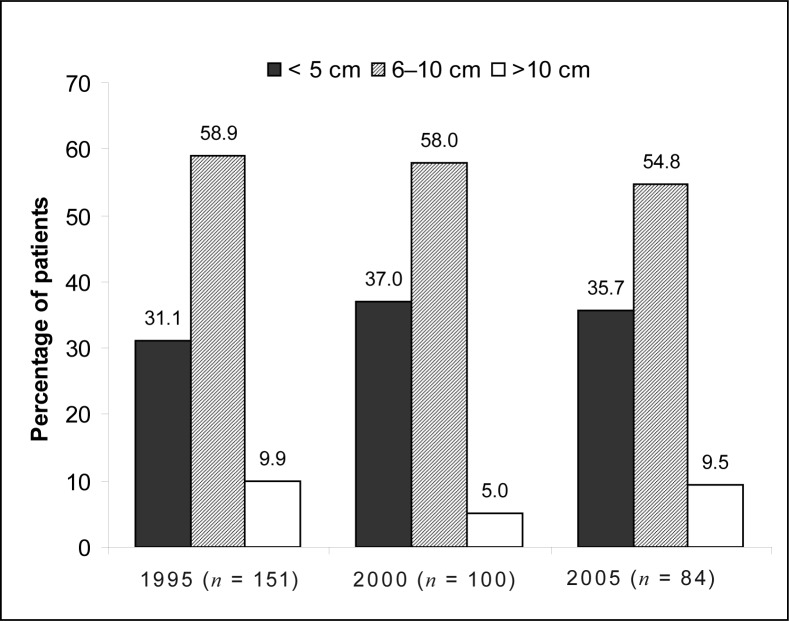
Length of the tumour in oesophageal carcinoma in 1995, 2000 and 2005

The length of the tumour was either unknown or not indicated in the patients’ files in a total of 139 cases – 31.7%, 32% and 20.8% of patients in 1995, 2000 and 2005 respectively. The results of the tumour length in cases where this information was available are shown in Figure 3.

**FIGURE 4:**
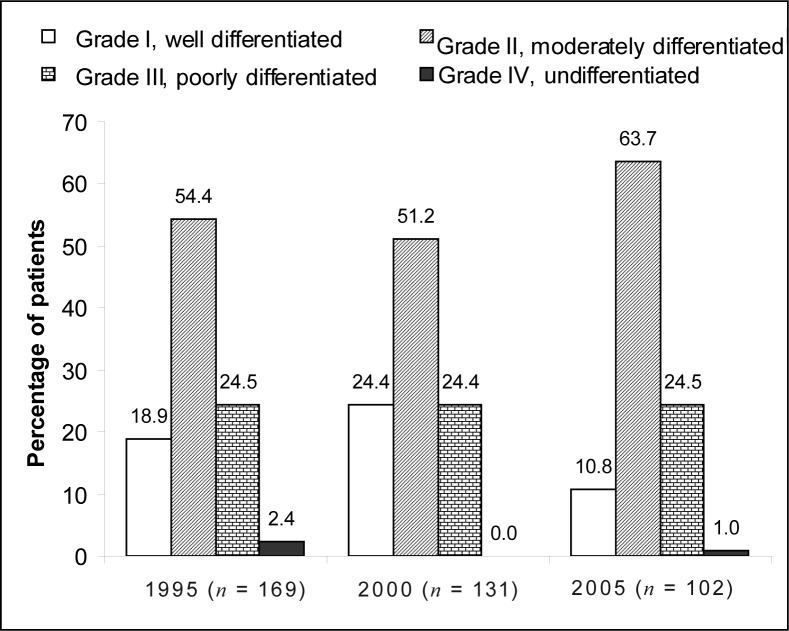
Histological grade of tumours in oesophageal cancer in 1995, 2000 and 2005

Figure 4 shows the distribution of the different histological grades of tumours in oesophageal carcinoma between 1995, 2000 and 2005. The histological grade of the tumour was unknown in 72 patients – 23.5%, 10.9% and 3.8% of patients in 1995, 2000 and 2005, respectively. More than 50% of patients in each of these years were diagnosed with Grade II, moderately differentiated tumours.

**FIGURE 5:**
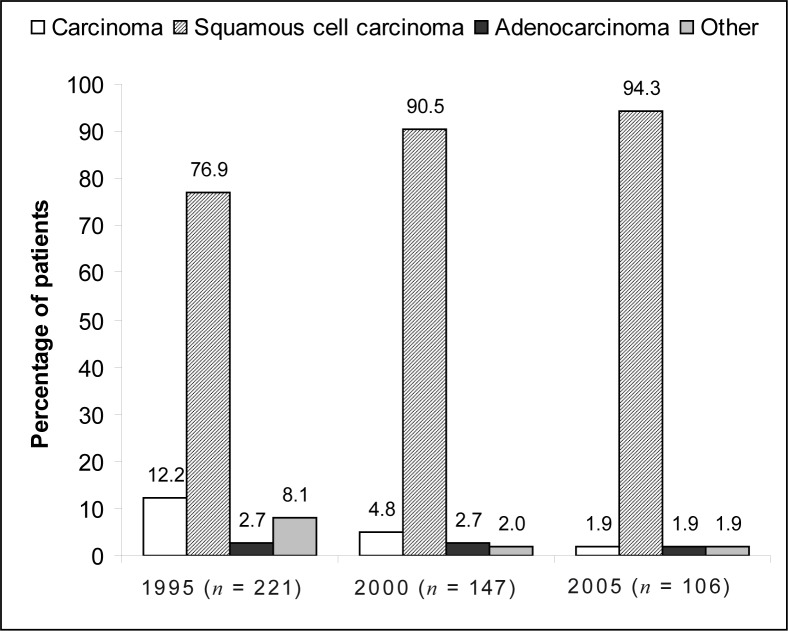
Distribution of different morphological types of oesophageal carcinoma in 1995, 2000 and 2005

With regard to the morphological type of oesophageal carcinoma, the majority of patients were diagnosed with squamous cell carcinoma – 76.9% in 1995, 90.5% in 2000 and 94.3% in 2005 (Figure 5). In a small number of patients, the specific morphological type was either not indicated (i.e. indicated only as ‘carcinoma’ in the patient file) or indicated as ‘other’. This occurred more often in the patient files from 1995.

Information regarding the development of fistulae in patients with oesophageal carcinoma was not available in 268 (56.5%) cases – 47.5% in 1995, 62.6% in 2000 and 67% in 2005. The majority of patients for whom information was available, however, did not develop fistulae, with this complication being observed in 20.7% of patients in 1995, 43.6% in 2000 and 31.4% in 2005. In 319 (67.3%) cases (52% in 1995, 83% in 2000 and 77.4% in 2005), no information regarding the development of sinuses was indicated in the patients’ files. Of those for whom information was available, 28.3% developed sinuses in 1995, 60% in 2000 and 42.7% in 2005.

Metastasis occurred in less than 40% of the patients for whom information was available (*n* = 180; 38.0%), with 37.7% in 1995, 31.3% in 2000 and 29.8% in 2005. In cases where metastasis occurred, the lungs were involved in 43% and lymph nodes in 31% of patient cases.

Of the 389 (82.1%) patients for whom information was available, more than 90% reported weight loss – 96.1% in 1995, 93.3% in 2000 and 96.7% in 2005. In each year, more than 85% of patients were diagnosed with Grade I dysphagia, with 89.6% in 1995, 86.4% in 2000 and 94.3% in 2005; 9.6%, 10.2% and 1.9% of patients did not experience any dysphagia in these three years respectively. These differences between 1995 and the other two years were statistically significant (*p* = 0.0022).

Based on the Eastern Cooperative Oncology Group (ECOG) performance status of patients with malignant diseases, less than 20% of the 330 patients for whom this information was available were evaluated as Grade 0, that is, they were fully active and able to carry out all pre-disease activities without restriction.^11^ Results for ECOG performance status grading of the patients investigated in 1995, 2000 and 2005 are summarised in Table 1.

**TABLE 1:** Grading of Eastern Cooperative Oncology Group (ECOG) performance status of patients diagnosed with oesophageal cancer in 1995, 2000 and 2005

Grade	Description	Percentage of patients		
		
		1995 (*n* = 161)	2000 (*n* = 103)	2005 (*n* = 66)
0	Fully active, able to carry out all pre-disease performance without restriction.	14.9	18.5	3
I	Restricted in physically strenuous activity, but ambulatory and able to carry out work of light or sedentary nature, e.g. light house work, office work.	36	30.1	43.9
II	Ambulatory and capable of self-care, but unable to carry out any work activities; up and about more than 50% of waking hours.	27.3	27.2	25.8
III	Capable of limited self-care, confined to bed or chair more than 50% of waking hours.	14.9	20.4	22.7
IV	Completely disabled – cannot carry out any self-care, totally confined to bed or chair.	6.8	3.9	4.6

**FIGURE 6:**
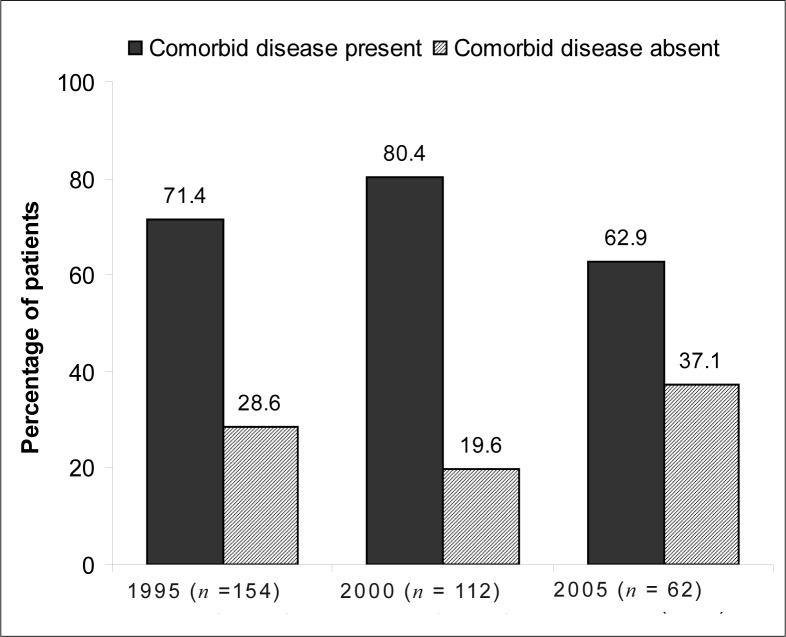
Percentage of patients with co-morbidity in addition to oesophageal cancer in 1995, 2000 and 2005

Information regarding the presence of co-morbid disease was available for 328 (69.2%) patients. The most commonly reported co-morbidity was respiratory disease, which occurred in 28.2% of the patients for whom information was available in 1995, 45.6% in 2000 and 28.7% in 2005 (Figure 6).

Information regarding the latest cancer status of these patients was available in 41.6% (*n* = 197) of the files. Of these, progression of the disease was noted in 51.9% of patients in 1995, 45.5% in 2000 and 69.2% in 2005.

The development of complications following therapy was unknown for 75.1% of the total group of patients. By the time of completion of the study, 65% of both the 1995 and 2005 patients had not returned for any follow-up visit, as opposed to 36% of the 2000 group of patients. Of those for whom follow-up information was available, 62% of the 2000 group of patients were deceased, compared to 30% of both the 1995 and 2005 patients.

## DISCUSSION

The decrease of 52% in the number of newly diagnosed cases of oesophageal carcinoma, from 221 cases in 1995 to 106 cases in 2005, could be attributed to several possible reasons. A possible explanation could be that, with the emergence of the HIV/AIDS epidemic in southern Africa, many potential patients died of HIV/AIDS-related diseases before they reached the age at which oesophageal cancer is most commonly diagnosed, which is approximately 60 years of age.^12, 13^ Most HIV/AIDS-related deaths in South African adults occur between 25 and 44 years of age.^14^ Additionally, the number of patients from other provinces, for example, the Eastern Cape, who previously visited National Hospital, could have decreased due to health policies preventing patients from one province visiting health care facilities in a neighbouring province. Insufficient primary health care, with poor diagnosis and referral of oesophageal carcinoma cases from clinics, could also have contributed to the decrease in the number of cases reported at the National Hospital from 1995 to 2005.

The racial distribution of oesophageal cancer remained fairly constant for the years 1995, 2000 and 2005 and reflected the general composition of the South African population.^15^ This observation could also be attributed to the fact that more Black patients visit National Hospital, especially the previously disadvantaged who could not afford medical aid as a result of historic disparities. Squamous cell carcinoma of the oesophagus was the most prevalent morphological type in all three years, and it could be concluded that this type of oesophageal cancer most commonly occurs in Black patients.

The gender distribution of patients with oesophageal cancer remained fairly constant for the years 1995, 2000 and 2005. The slight increase in the percentage of female patients in 2005 could possibly be attributed to an increase in tobacco^16^ and alcohol consumption^17^ in women in general over this period of time.

With regard to the histological type of oesophageal cancer, squamous cell carcinoma increased steadily. The percentage of cases presenting with adenocarcinoma remained constant at less than 3% for the years 1995, 2000 and 2005. These findings are similar to results published by Cherian et al.,^18^ who reported squamous cell carcinoma in 92% of patients with oesophageal malignancy in India between 1989 and 2004. Contrary to these findings, several studies reported an increase in the number of patients diagnosed with adenocarcinoma of the oesophagus.^19, 20, 21, 22^

Dysphagia is a common symptom in oesophageal cancer and was observed in more than 85% of the study population. Weight loss, which is directly associated with dysphagia, occurred in more than 90% of patients. However, it should be kept in mind that the treatment of malignancies and the disease process could also contribute to physical deterioration.

Due to the retrospective nature of the study, information regarding certain aspects of the disease was lacking in many patient files. The reliable interpretation of some results was thus not possible. For example, patients could have died from local disease before extra-oesophageal extension of the tumour (indicated in only 49 [10.3%] patient files) and metastasis of the malignancy (indicated in 180 [38.0%] files) could be diagnosed. In addition, only 5% of patients with oesophageal carcinoma develop fistulae or sinuses,^2^ which could explain the high number of patient files not containing information regarding these complications.

## CONCLUSION

The findings of this study showed that, for the years 1995, 2000 and 2005, the majority of patients with oesophageal cancer were Black middle-aged to elderly males who were diagnosed with squamous cell carcinoma. Although an increase in the number of cases presenting with squamous cell carcinoma of the oesophagus was noted, other variables, such as race, age and gender, remained constant for these three years.
